# The Potential Impact of Long-Acting Cabotegravir for HIV Prevention in South Africa: A Mathematical Modeling Study

**DOI:** 10.1093/infdis/jiaa296

**Published:** 2020-06-03

**Authors:** Jennifer A Smith, Geoffrey P Garnett, Timothy B Hallett

**Affiliations:** 1 MRC Centre for Global Infectious Disease Analysis, Department of Infectious Disease Epidemiology, Imperial College London, London, United Kingdom; 2 Bill and Melinda Gates Foundation, Seattle, Washington, USA

**Keywords:** heterosexual transmission, HIV prevention, long-acting cabotegravir, mathematical models, South Africa

## Abstract

**Background:**

Although effective, some oral pre-exposure prophylaxis (PrEP) users face barriers to adherence using daily pills, which could be reduced by long-acting formulations. Long-acting cabotegravir (CAB LA) is a potential new injectable formulation for human immunodeficiency virus (HIV) PrEP being tested in phase III trials.

**Methods:**

We use a mathematical model of the HIV epidemic in South Africa to simulate CAB LA uptake by population groups with different levels of HIV risk. We compare the trajectory of the HIV epidemic until 2050 with and without CAB LA to estimate the impact of the intervention.

**Results:**

Delivering CAB LA to 10% of the adult population could avert more than 15% of new infections from 2023 to 2050. The impact would be lower but more efficient if delivered to populations at higher HIV risk: 127 person-years of CAB LA use would be required to avert one HIV infection within 5 years if used by all adults and 47 person-years if used only by the highest risk women.

**Conclusions:**

If efficacious, a CAB LA intervention could have a substantial impact on the course of the HIV epidemic in South Africa. Uptake by those at the highest risk of infection, particularly young women, could improve the efficiency of any intervention.

## Teaser

Using injectable cabotegravir for HIV prevention could, if efficacious, be a valuable addition to the portfolio of HIV prevention options in South Africa and could have a substantial impact on the progression of the epidemic.

There continues to be a substantial number of new human immunodeficiency virus (HIV) infections worldwide, with an estimated 1.7 million people newly infected in 2018 [1]. The majority of these are in sub-Saharan Africa (58%), with more than half of those occurring in just 4 countries: South Africa, Mozambique, Nigeria, and Tanzania [2]. Incidence is particularly high among adolescent girls and young women (AGYW) aged 15–24 years in sub-Saharan Africa, who are 3 times more at-risk than their male counterparts [3–5].

Several HIV prevention methods are available, including male and female condoms, voluntary medical male circumcision (VMMC), early access to antiretroviral treatment (ART), and tenofovir/emtricitabine (TDF/FTC) daily oral pre-exposure prophylaxis (PrEP; termed “oral PrEP”). When these are implemented in combination, declines in HIV incidence can be observed at the population level [4]. However, in general, they have not been deployed widely enough to reach their full potential in reducing new HIV infections.

Oral PrEP has been a promising addition to the HIV prevention portfolio. When taken as prescribed, it is highly effective at reducing HIV acquisition risk [6, 7]. However, the need to take a daily pill can be a barrier to adherence for some users, and oral PrEP implementation projects have found high early drop-off rates among African AGYW [8, 9]. Long-acting formulations of PrEP, including rings, injections, and implants, which will likely have different adherence barriers, are in development [10].

In 2016, an intravaginal ring lasting 1 month and containing dapivirine was shown to be moderately effective at reducing HIV incidence, with higher effectiveness among women with evidence of product use, and is now undergoing regulatory review [11, 12]. However, user preference studies have shown a general preference for injectable forms over pills and rings, although these may not translate directly to real-life uptake if new PrEP modalities become available [13–15]. Parallels in the reproductive health sphere demonstrate the increase in total contraceptive use when a new contraceptive method becomes available [16].

One promising candidate for an injectable form of PrEP is long-acting cabotegravir (CAB LA). Cabotegravir (formerly GSK1265744) is a potent integrase strand transfer inhibitor with a high barrier to resistance that can be formulated as an oral tablet or a long-acting suspension for injectable intramuscular use [17]. This makes it a suitable candidate for use in both HIV treatment (in combination with a second long-acting injectable agent) and prevention.

Two phase IIa studies, ÉCLAIR (NCT02076178) and HPTN 077 (NCT02178800), demonstrated that injectable CAB LA for HIV prevention was well tolerated with an acceptable safety profile, despite more frequent injection site reactions among participants in the active arm compared to the placebo arm [18, 19]. A dosing regimen of 600 mg every 8 weeks consistently met pharmokinetic targets for both men and women in HPTN 077.

Phase IIb/III studies HPTN 083 (NCT02720094) and HPTN 084 (NCT03164564) are now underway to compare the safety and efficacy of CAB LA with daily oral PrEP among (1) HIV-uninfected cisgender men and transgender women who have sex with men (MSM and TGW) and (2) HIV-uninfected women [20, 21]. The HPTN 083 trial was stopped ahead of schedule in May 2020 and the results announced that CAB LA is highly effective compared to oral PrEP at reducing HIV incidence among MSM and TGW [22]. Results for HPTN 084 are anticipated in 2021.

Long-acting cabotegravir could, if efficacious, be a valuable addition to the HIV prevention portfolio, particularly in locations and among populations where HIV incidence remains high. South Africa has the highest number of annual new HIV infections of any country worldwide [2]. Annual incidence among 15- to 24-year-old women was 1.51% in 2017 compared to .79% among 15- to 49-year-old adults [5]. Oral PrEP has been available to some key populations, including young people at university and community testing sites, since 2016, but it has not been consistently rolled out to all those at the highest risk of HIV acquisition [23]. This analysis uses a mathematical model to explore the potential role of a new PrEP product in the context of the scale-up of existing HIV prevention products in South Africa, and it allows us illustrate the relationship between coverage and impact in different population groups.

## METHODS

We calibrated a deterministic compartmental simulation model to represent the ongoing HIV epidemic in South Africa over time using data on demography, age-specific and sex-specific HIV prevalence and incidence, ART and VMMC scale-up, and patterns of male condom use [5, 24]. The model has been described in detail elsewhere, and full details of the parameterisation and calibration are provided in the Supporting Information [25–27]. Key features of the model include representation of the population age structure (by fractions of a year), differences in sexual behavior by age (with respect to sex acts, condom use, partner change rates), the transmission of HIV through heterosexual sex, and the progression towards acquired immune deficiency syndrome, the ART cascade, and prevention interventions that are already in use. The model simulates the HIV epidemic from 1985 to 2050.

### Prevention Interventions

Four existing prevention interventions are simulated in the model. These are as follows: (1) condom use; (2) VMMC; (3) early ART; and (4) oral PrEP.

The historical expansion of these interventions is calibrated to available data. From 2019 onwards, we make 2 sets of assumptions about how these interventions continue to be used. This creates 2 counterfactual scenarios to use as baselines against which to introduce CAB LA (Table 1, with full details on each intervention in the Supporting Information). In the first, termed the “Constant Coverage” baseline, there is no additional scale-up of the existing prevention interventions described above—that is, condom use, VMMC, and early ART remain constant at current levels from 2019, and oral PrEP is not introduced (we assume that the current low levels of oral PrEP use have negligible impact on the epidemic). In the second “Projected Scale-up” baseline, we simulate the degree of long-term scale-up that might be anticipated on the basis of current planning. The predicted levels of each intervention were based on those previously proposed by Smith et al [26] and updated where appropriate (Table 1). The 2 counterfactual scenarios provide different epidemic contexts against which to estimate the impact of a CAB LA intervention, including any modifications due to the presence of other prevention methods.

**Table 1.** Summary of Assumed Scale-Up of Existing Prevention Interventions^a^

				Effective Coverage in Main Target Group	
Prevention Intervention	Efficacy	Available From	Main Target Group(s)	Constant Coverage	Projected Scale-Up
Male condoms	90%	2019	FSW	29%	60%
VMMC	60%	2019	Young men	43%	60%
Early ART	85%	2019	All	40%	60%
Oral PrEP	90%	2019	FSW High-risk young women	0% 0%	10% 5%

Abbreviations: ART, antiretroviral treatment; FSW, female sex workers; PrEP, pre-exposure prophylaxis; VMMC, voluntary medical male circumcision.

^a^Efficacy refers to the protection afforded by perfect use of a product. Effective coverage is the proportion of people who fully adhere to a product such that they benefit from its protection. Full details of each intervention implementation are available in the Supporting Information.

### Roll Out of Long-Acting Cabotegravir

The model simulates the uptake of CAB LA by HIV-negative adults under 5 scenarios: (1) adults (aged 15–49 years, irrespective of HIV risk); (2) men (aged 15–49 years, irrespective of HIV risk); (3) women (aged 15–49 years, irrespective of HIV risk); (4) high-risk women (aged 15–49 years, including female sex workers [FSWs]); and (5) high-risk young women (aged 15–30 years, plus 15- to 49-year-old FSWs).

Table 2 gives the profiles of each of these groups. “High-risk” refers to the women’s assigned risk strata within the model, with the size and sexual behavior of the groups being selected as part of the calibration procedure (within prespecified bounds). This results in 10% of all women being initially classed as high-risk. We assume that women can receive CAB LA based on their level of HIV risk, but not men, because there is a smaller difference in HIV incidence between “low-risk” and “high-risk” groups for men compared with women in the model. This is calculated from the combination of input parameters that determine sexual behavior and the data to which the model was calibrated. For scenarios (4) and (5), we assume that there is also some misspecification of risk perception such that there is one-tenth CAB LA coverage among the equivalent low-risk women for each of the target groups and at every coverage level (eg, when there is 20% coverage among 15- to 30-year-old high-risk women, there is also 2% coverage among 15- to 30-year-old low-risk women).

**Table 2.** User Profiles for Focus of Long-Acting Cabotegravir Scale-Up^a^

			Mean HIV Incidence in 2020 (Per 100 Person-Years)		
CAB LA Users	Age (Years)	Size of Risk Group in 2020	Constant Coverage Baseline	Projected Scale-Up Baseline	Other Key Characteristics
1. Adults	15–49	23.8 million	0.9	0.9	
2. Men	15–49	12.6 million	0.7	0.6	
3. Women	15–49	11.2 million	1.2	1.2	Includes FSW
4. High-risk women	15–49	770 000	3.1	2.9	Includes FSW
5. High-risk young women	15–30	550 000	3.3	3.1	Includes 15- to 49-year-old FSW

Abbreviations: CAB LA, long-acting cabotegravir; FSW, female sex workers; HIV, human immunodeficiency virus.

^a^All recipients of CAB LA are assumed to be HIV-negative. HIV incidence is lower in the Projected Scale-up baseline because existing prevention interventions are scaled up from 2019.

We simulate CAB LA uptake by each population group to achieve “effective coverage” levels of 1%, 5%, 10%, and 20%. Effective coverage is defined as the proportion of users who benefit from full adherence to the product. Thus, persons that have received the product but do not use it sufficiently to achieve effectiveness are classed outside this coverage value.

Long-acting cabotegravir introduction is simulated in the model from 2023. Scale-up is linear and takes 4 years to achieve the specified effective coverage level. We assume that (1) CAB LA has 90% efficacy at reducing the per-sex-act risk of HIV acquisition for users, (2) a CAB LA injection is given to users every 8 weeks, and (3) the mean duration of continuous CAB LA use is 5 years. We perform a sensitivity analysis reducing CAB LA efficacy to 60% and the mean duration of use to 1 year. When CAB LA is introduced into the Projected Scale-up baseline, we assume that all uptake is among new PrEP users (ie, CAB LA is additive to oral PrEP), based on the observation that in the family planning sphere, addition of a new contraceptive method generally expands the overall method usage [16].

We simulate the population of South Africa from 2020 to 2050, and we estimate the number of HIV infections averted by CAB LA, if safe and efficacious, from its introduction in 2023 over 5, 10, and 25 years, compared with both the Constant Coverage and Projected Scale-up baselines. The number of infections averted represents new HIV infections among all 15- to 49-year-old adults and includes indirect infections. We report the person-years (PY) spent on CAB LA and the number of CAB LA injections dispensed in these periods. We calculate the number of PY on CAB LA required to avert 1 HIV infection by dividing the total PY of CAB LA dispensed in a specified time period by the total HIV infections averted in that period (this metric is akin to the “number needed to treat” (NNT) concept of Buchbinder et al [28] for oral PrEP). All results are presented without discounting. Ethical approval and the consent of participants were not required for this mathematical modeling study.

## RESULTS

**Figure 1.**
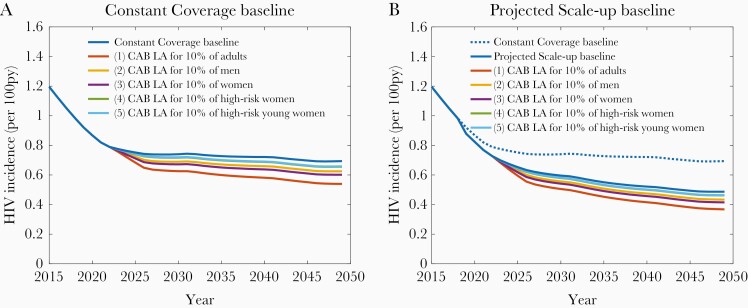
Projected human immunodeficiency virus (HIV) incidence among adults aged 15–49 years from 2015 to 2050. Long-acting cabotegravir (CAB LA) is delivered to 10% of each population group. py, person-years. A, Constant Coverage baseline; B, Projected Scale-up baseline.

Figure 1 shows the projected HIV incidence for both the Constant Coverage and Projected Scale-up baselines together with the potential impact on HIV incidence that can be achieved by the uptake of CAB LA by 10% of each population group (scenarios 1–5). Under the Constant Coverage baseline with no CAB LA intervention, HIV incidence among 15- to 49-year-old adults is .9% in 2020 and declines slightly to level off at .7%. Under the Projected Scale-up baseline, HIV incidence declines further to .5% by 2050. The introduction of CAB LA could have a substantial impact under both baselines, with the magnitude of the impact dependent on both the size of each user group and the proportion of ongoing HIV infections occurring within it. Uptake of CAB LA by 10% of high-risk women (scenario 4) or 10% of high-risk young women (scenario 5) can decrease HIV incidence by 5% by 2050 in both the Constant Coverage baseline and the Projected Scale-up baseline. Thus, there is only a small marginal benefit of CAB LA use among high-risk women of all ages compared with high-risk younger women at this uptake level.

Use of CAB LA by 10% of all women without prioritizing by HIV risk (scenario 3) can reduce HIV incidence by 13% against the Constant Coverage baseline and 15% against the Projected Scale-up baseline, and by 10% and 11%, respectively, when delivered to men (scenario 2). If all adults are eligible to receive CAB LA (scenario 1), HIV incidence could decline by 22% and 24% by 2050 compared with each baseline, when used by 10% of the population. This represents averting 15% of incident infections from 2023 to 2050 for both baselines. The same patterns of results are seen at 1%, 5%, and 20% uptake levels for each population group, with the reductions in incidence scaled accordingly (Figure S9 in Supporting Information). With a lower assumed efficacy (60% per sex act) and a shorter mean duration of use (1 year), the proportion of new infections averted from 2023 to 2050 is reduced from 15% (as reported above) to 10% if CAB LA is used by 10% of all adults, and from 3% to 2% if CAB LA is used by 10% of high-risk young women (Figure S10 in Supporting Information).

**Figure 2.**
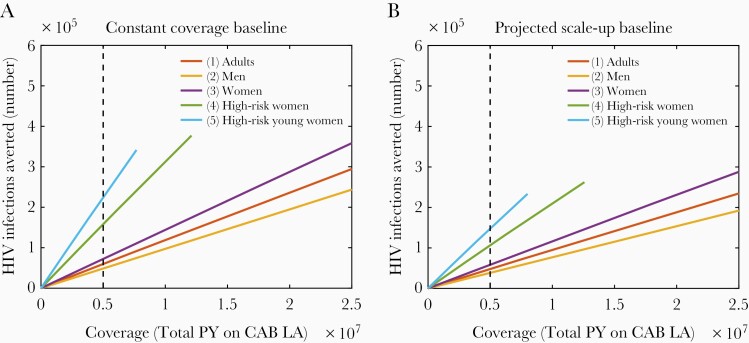
Number of HIV infections averted after the introduction of long-acting cabotegravir (CAB LA) to different population groups. The CAB LA is distributed from 2023 to 2050. Dashed line marks 5 million person-years of CAB LA dispensed. A, Constant Coverage baseline; B, Projected Scale-up baseline.

Figure 2 shows the total number of HIV infections averted from 2023 to 2050 after the introduction of CAB LA to each of the population groups, against the total PY of CAB LA delivered in that time. The extent of CAB LA coverage in PY (span on x-axis) is determined by the size of the population group in question. The extent of CAB LA impact (span on y-axis) is determined by the number of infections occurring within the population group plus subsequent onward transmissions. The gradient of the coverage-impact line represents a measure of the efficiency of the intervention, and it shows that the same number of CAB LA injections give the greatest impact when used by high-risk young women (scenario 5), followed in order of declining impact by high-risk women (scenario 4), all women (scenario 3), adults (scenario 1), and men (scenario 2). The dashed line shows, as an example, that 5 million PY of CAB LA injections (representing 33 million injections) can avert more than 3 times as many infections when used by high-risk young women compared with men (also see Table 3). The time taken to dispense 5 million PY will vary between the population groups depending on the size of the group.

**Table 3.** Number of HIV Infections Averted Over Thirty Years by Dispensing Five Million Person-Years of Long-Acting Cabotegravir

CAB LA Users	Constant Coverage Baseline	Projected Scale-Up Baseline
1. Adults	60 000	48 000
2. Men	49 000	38 000
3. Women	72 000	58 000
4. High-risk women	160 000	110 000
5. High-risk young women	220 000	150 000

Abbreviations: CAB LA, Long-acting cabotegravir; HIV, human immunodeficiency virus.

A comparison of Figure 2A and B shows that the overall impact of CAB LA in terms of absolute numbers of infections is lower in the Projected Scale-up baseline than the Constant Coverage baseline. This is because the expansion of other prevention interventions already reduces HIV incidence, so there are fewer ongoing infections available for CAB LA to avert. Furthermore, the marginal impact and efficiency of greater use among higher risk women is lessened when other interventions are being scaled up.

**Figure 3.**
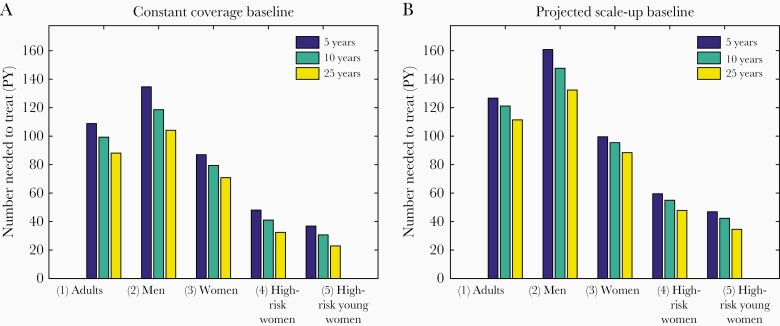
The number of person-years (PY) of long-acting cabotegravir required to avert 1 HIV infection. Calculation performed assuming 10% coverage in each population group. A, Constant Coverage baseline; B, Projected Scale-up baseline.

Figure 3 shows the number of PY of CAB LA required to avert 1 HIV infection when delivered to different population groups. This calculation is performed at 10% uptake of CAB LA by each population group, but the results are robust to different coverage levels (Figure S11 in Supporting Information). Under the Projected Scale-up baseline, when CAB LA is used by high-risk young women only (scenario 5), 1 HIV infection is averted by 47 PY of use over a 5-year time frame, or 35 PY of CAB LA over a 25-year time frame. This compares to 100 and 88 PY of use, respectively, if CAB LA is given to women irrespective of risk (scenario 3), and 127 and 111 PY for all adults (scenario 1).

Fewer PY of CAB LA use are required to avert 1 infection when calculated over a longer time frame because the initial roll-out period, when CAB LA has not yet reached full coverage, contributes less compared with the total duration. The Constant Coverage baseline shows the same pattern but with fewer PY of CAB LA required to avert 1 infection, because overall incidence is higher (Figure 3B).

The future cost of CAB LA is unknown, but by assuming different costs we can gain an indication of the potential cost of averting 1 HIV infection based on the PY of CAB LA required. For example, at a total cost (including procurement and delivery) of $300 per year, it would cost $38 000 to avert 1 infection if CAB LA is used by all adults, or $14 000 to avert 1 infection if CAB LA is used only by high-risk young women. If the total cost were $1000 per year, the cost of averting 1 infection would be $127 000 and $47 000, respectively.

## DISCUSSION

We have evaluated the potential impact of introducing a long-acting PrEP product, parameterized to represent injectable cabotegravir, to different population groups in South Africa, against a backdrop of either little or extensive scale-up of other prevention technologies. We show that a CAB LA intervention, if the product is efficacious, could have a substantial impact on the course of the HIV epidemic, averting 15% of new infections from 2023 to 2050 when delivered to 10% of the adult population. Use of CAB LA by those at higher risk of infection, particularly young women, could improve the efficiency of any intervention, as measured by the NNT. For example, 127 PY of use are needed to avert 1 infection when used by adults compared to 47 when used by high-risk young women when introduced alongside the scale-up of other prevention technologies. These high-risk groups have a baseline incidence of approximately 3% per PY (Table 2), which approximates the World Health Organization (WHO) recommendation for oral PrEP use by those at “substantial risk of infection” [29].

We have provided a rough illustration of how the impact and efficiency of a CAB LA intervention will influence its cost-effectiveness. To fully understand the implications requires consideration of the value of an infection averted, which may vary by location and population.

Long-acting injectable PrEP for HIV prevention is a promising potential addition to the range of existing prevention options. Although the WHO recommends that oral PrEP should be available to populations at substantial risk of HIV infection, defined as incidence of more than 3%, adherence can be a challenge for some users and current use is limited [8, 23, 29]. Long-acting PrEP formulations, such as CAB LA, could remove some adherence barriers faced by some oral PrEP users and enable more individuals at risk of HIV infection to access the benefits of PrEP overall. Nonetheless, CAB LA users may face different adherence barriers to oral PrEP, and the implications of nonadherence to a long-acting agent are likely to be different, more complicated, and potentially more severe than nonadherence to daily use agents [30]. In addition, there is potential for the emergence of drug resistance if new infections occur during the long observed pharmacokinetic tail phase, which may need protection with daily oral PrEP [31].

Ultimately, combination prevention approaches are likely required to meet the needs of all at-risk individuals, and this may include other novel forms of PrEP such as the dapivirine ring, and eventually antiretroviral implants, as well as CAB LA, oral PrEP, and other existing and future technologies. Some of the challenges of introducing oral PrEP have been to foster demand for the product and then to support continued use with high adherence after initiation, particularly among AGYW [9]. Some lessons learned from oral PrEP regarding uptake and persistence may also be relevant to CAB LA and other new products.

Although we find that CAB LA use by those at highest risk of HIV infection is the most efficient way to implement an intervention, our ability to identify those “at risk” in reality may be limited. Women, and particularly AGYW, may not perceive themselves as at risk [32]. However, clinical trials have succeeded in recruiting participants with high HIV incidence—for example, the recent ECHO trial reported HIV incidence of 3.81% in its control arm—and some correlates of risk have been identified in oral PrEP studies [32, 33]. Contrarily, considerations of equity and acceptability may make it unrealistic to provide CAB LA to individuals based on risk alone—for example, some may not meet specific risk criteria per se but instead may be unable to use alternative prevention methods.

There are several limitations to this analysis. The major limitation to the modeling approach is the lack of uncertainty in the input epidemiological parameters. Instead, we have parameterized the model with the most recent data available and performed scenario analyses with 2 baselines and varying coverage levels. Uncertainty over costs, delivery channels, user acceptability, and other interventions over a 30-year period are greater than uncertainty in many model parameters, so we have chosen to illustrate scenarios that highlight some key relationships between coverage and impact. Sensitivity analyses show how the quantitative results are moderated by the assumed efficacy and duration of CAB LA use, but the qualitative results hold after minor adjustments to the input parameters, and it is these that are worth emphasising.

We have focused on South Africa, but there are many other regions with HIV incidence among particular geographies or key populations for whom CAB LA might also be beneficial. The model does not include MSM or TGW because the burden of the HIV epidemic in South Africa is overwhelmingly among the heterosexual population. Furthermore, there is little data among risk group size and behavior with which to reliably parameterize this model. However, these groups are known to be at very high risk of HIV infection, and their inclusion in any intervention programming would be essential. HIV prevalence of 18.1% has been estimated among MSM in South Africa [2], and HIV incidence was 9% and 14% among MSM and TGW in Soweto and Cape Town, respectively, in HPTN 075 [34].

Health economic modeling is required to elucidate further the contexts in which CAB LA may or may not be cost-effective. Two early studies found long-acting injectable PrEP very cost-effective or cost-saving if prioritized to those at highest risk of infection in South Africa [35, 36]; another recent study shows that injectable PrEP is not cost-effective when delivered to heterosexual men in South Africa [37].

## CONCLUSIONS

The introduction of CAB LA could be a valuable addition to the portfolio of HIV prevention options in South Africa and could have a substantial impact on the progression of the epidemic. The efficiency of any intervention, which will determine its cost-effectiveness, will be determined by the ability to identify and support use by those at the highest risk of infection.

## Supplementary Data

Supplementary materials are available at *The Journal of Infectious Diseases* online. Consisting of data provided by the authors to benefit the reader, the posted materials are not copyedited and are the sole responsibility of the authors, so questions or comments should be addressed to the corresponding author.

jiaa296_suppl_Supplementary_Material

## Notes

***Acknowledgments*.** We thank Mary Aikenhead, who provided helpful input on the design of the study.

***Author contributions.*** J. A. S., G. P. G., and T. B. H. designed the preliminary analysis. J. A. S. coded the model, produced the results, and drafted the manuscript. J. A. S., G. P. G., and T. B. H. revised the manuscript. All authors have read and approved the final manuscript.

***Financial support.*** This work was funded by the Bill & Melinda Gates Foundation (OPP1194416). J. A. S. and T. B. H. also acknowledge funding from the Medical Research Council Centre for Global Infectious Disease Analysis (MRC GIDA) (MR/R015600/1); this award is jointly funded by the UK Medical Research Council (MRC) and the UK Department for International Development (DFID) under the MRC/DFID Concordat agreement and is also part of the EDCTP2 programme supported by the European Union.

***Potential conflicts of interest.*** J. A. S. reports grants and personal fees from the Bill & Melinda Gates Foundation (BMGF) during the conduct of the study and grants from the United States Agency for International Development (USAID) and the BMGF outside the submitted work. G. P. G. is an employee of the BMGF. T. B. H. reports grants and personal fees from the BMGF during the conduct of the study; grants from USAID, BMGF, World Bank, the Joint United Nations Programme on HIV/AIDS, Rush Foundation, and Wellcome Trust outside the submitted work; and personal fees from BMGF, New York University, World Health Organization, and Global Fund to Fight AIDS, TB and Malaria (GFATM) outside the submitted work. All authors have submitted the ICMJE Form for Disclosure of Potential Conflicts of Interest. Conflicts that the editors consider relevant to the content of the manuscript have been disclosed.
