# Supplementary material for: The Potential Impact of Long-Acting Cabotegravir for HIV Prevention in South Africa: A Mathematical Modeling Study
Source: J Infect Dis. 2020 Jun 3;224(7):1179–86. doi: 10.1093/infdis/jiaa296 (PMC8514192; doi:10.1093/infdis/jiaa296)
Supplement: jiaa296_suppl_Supplementary_Material [file jiaa296_suppl_supplementary_material.docx]

**The potential impact of long-acting cabotegravir for HIV prevention in South Africa: a mathematical modelling study**

SUPPORTING INFORMATION

**Authors:** Jennifer A. SMITH^1^, Geoffrey P. GARNETT^2^, Timothy B. HALLETT^1^

^1^MRC Centre for Global Infectious Disease Analysis, Department of Infectious Disease Epidemiology, Imperial College London, UK

^2^Bill and Melinda Gates Foundation, Seattle, WA, USA

**Model design**

The mathematical model is designed to represent heterosexual HIV transmission at the population level in South Africa, a mature generalised HIV epidemic. The model population is divided into compartments that are distinguished by sex, age, infection stage, sexual behaviour and exposure to different types of interventions, with events (e.g. HIV infection, death, ART initiation, etc.) represented as movement between these compartments.[1-3] The model is population-based and deterministic; individuals and partnerships are not explicitly tracked, and the results hold for large populations only.

In the model, the natural history of HIV infection is represented by six consecutive compartments, each relating to a different stage of HIV infection. Individuals becoming infected move from the ‘Susceptible’ compartment to progress through these six consecutive compartments: ‘Acute Infection’, a subsequent stage when CD4 count is above 350 cells per microliter, a stage when CD4 count is between 350 and 200 cells per microliter, an ‘AIDS (early)’ stage, an ‘AIDS (advanced)’ stage and lastly, an ‘AIDS (severe)’ stage. Infectiousness of infected individuals varies over the course of infection, with a peak of infectiousness in the ‘Acute infection’ phase and a period of heightened infectiousness in the ‘AIDS (advanced)’ phase.[4]

Heterogeneity in sexual behaviour is incorporated in the model by stratifying men and women into risk groups defined by their mean partner change rate. The distribution across these strata and the mean partnership change rates for men and women are estimated through calibrating the model to age-specific HIV prevalence and incidence data from South Africa (see Model Calibration section below).[5]

HIV transmission occurs through heterosexual sexual partnerships. Although these are instantaneous in the model, the influence of variation in duration of partnership is reflected by specifying the rate of infection through the partnership as a function of the number of sex acts in partnerships per year. That is, short-term partnerships effectively have a low number of sex acts in total, whilst partnerships maintained over a longer time period would have a higher number of sex acts. Those in the higher risk groups tend to form more partnerships, but each of these partnerships is of a shorter duration and comprises fewer sex acts and higher condom use. The probability of HIV transmission per sex act for those in the chronic stage of infection is based on a meta-analysis,[6] with the relative infectiousness according to stage of infection based on data from serodiscordant heterosexual couples in sub-Saharan Africa.[4, 7]

There are four opportunities to initiate ART, specified with different initiation rules. Treatment can be initiated an average of one year after infection (i.e. as soon as infection will be detected on average in an intensive programme), or when an individual’s CD4 count drops below 350, 200, or 50 cells per microliter. In these analyses, ‘Early ART’ refers to the first criterion (i.e. initiated an average of one year after infection) while ‘Late ART’ refers to the latter three (Figure S1). These are used to represent the pattern of actual ART initiation in recent years in South Africa.[8] Individuals initiating ART when their CD4 count drops below 200 do so after a certain waiting time. Due to clinical need, no waiting time is assumed for individuals who are put on treatment when their CD4 drops below 50. Drop outs from the ‘Late ART’ category progress to the AIDS (severe) stage after a period of slightly heightened infectiousness represented by the ‘ART drop-out’ compartment (Figure S1). ART is assumed to extend the survival of treated individuals (depending on whether ART is initiated ‘early’ or ‘late’) while reducing their infectiousness.[7, 9]

Figure S1. Representation of HIV natural history and ART initiation

The impact of condom use is to reduce the chance of transmission in the sex acts in which they are used. There are therefore two parameters specifying the impact of condom use:

(1) Efficacy in preventing transmission in a sex act if they are used correctly

(2) Usage in sex acts - proportion of sex acts in a partnership in which they are used (this can vary by partnership type).

Usage changes over time to reflect the increase in condom use in South Africa. Repeated cross-sectional surveys indicate that, reported condom use at last sex increased from 27% in 2002 to 36% in 2012.[10]

The influence of male circumcision is represented by dividing the male population into two categories, circumcised and uncircumcised. Circumcised men are less likely to acquire HIV infection by a fixed multiplicative factor per sex act with HIV-infected women. The probability of transmission of infection per sex act is assumed to be the same from both circumcised and uncircumcised men to women. The proportion of men in the model starting sex that enter the circumcised group corresponds to the proportion of men that are circumcised at birth or during adolescence.[11]

Age-specific fertility and mortality rates over time are taken directly from the ASSA2008 model.[12]

**Model calibration**

The model was using originally calibrated using sum of squares for a number of parameters – namely the underlying per sex act transmission probability, risk group sizes and behavioural parameters (Table S1). These were fit to match the model outputs to several data sources:

(1) HIV prevalence of South African adults aged 15+, scaling data from the national antenatal clinic (ANC) survey, as performed by Granich and colleagues.[13, 14]

(2) HIV prevalence data from 2002-2012 for adults aged 15-49 from the HSRC 2012 National Survey.[10]

(3) HIV incidence data from 2002-2012 for adults aged 15-49 from the HSRC 2012 National Survey.[10]

(4) Age- and sex-specific prevalence in 2012 for adults aged 15-49 from the HSRC 2012 National Survey.[10]

(5) Age-specific incidence for Kwa-Zulu Natal, 2003-2011, adjusted to match mean incidence to the estimate for South Africa.[15, 16]

The calibration was then assessed by hand for an acceptable fit to the more recent HSRC 2017 National HIV Survey and 2016 Demographic Health Survey (DHS) for South Africa.[5, 17]

Table S1. Fitted model parameters

| **Parameter** | **Fitted value (South Africa)** |
| --- | --- |
| HIV transmission probability per sex act | 3.52 x 10^-4^ |
| Degree of assortativity in sexual mixing with respect to risk status | 0.0586 |
| Degree of assortativity in sexual mixing with respect to age | 0.593 |
| Proportion in [low high FSW] risk group (women) | [0.900 0.0740 0.0260] |
| Proportion in [low medium high] risk group (men) | [0.817 0.169 0.0143] |
| Mean partner change rate for low risk group (women) | 0.287 |
| Mean partner change rate for low risk group (men) | 1.03 |
| Multiplicative factor for partner change rate of high risk group (women) | 19.2 |
| Multiplicative factor for partner change rate of FSW (women) | 36.9 |
| Multiplicative factor for partner change rate of medium risk group (men) | 2.15 |
| Multiplicative factor for partner change rate of high risk group (men) | 108 |
| Multiplicative factor for 5-year age groups (women): 15-19, 20-24, 25-29, 30-34, 35-39, 40-44, 45-49, 50-54 years | [1.72 2.92 4.09 1.86 1.57 1.17 1.18 0.87] |
| Multiplicative factor for 5-year age groups (men): 15-19, 20-24, 25-29, 30-34, 35-39, 40-44, 45-49, 50-54 years | [2.36 4.80 4.38 1.15 2.10 0.87 2.02 0.10] |
| Initial condom use by low risk group | 3.20 x 10^-3^ |
| Multiplicative factor for initial condom use by medium risk group (men) and high risk group (women) | 1.36 |
| Multiplicative factor for initial condom use by high risk group (men) and FSW (women) | 2.60 |

Figure S2. Model fit to South African demography data

Figure S3. Model calibration to South Africa HIV prevalence and incidence

Figure S4. Model fit to age-specific prevalence over time

Figure S5. Model fit for age- and sex-specific prevalence in 2012

Figure S6. Model fit for age- and sex-specific incidence, 2007

Figure S7. Model fit for ART coverage

Figure S8. Model fit for uptake of voluntary medical male circumcision

**Existing prevention interventions**

The existing prevention interventions are incorporated into the model by dividing the population into different strata representing those receiving the intervention and those not receiving it. The interventions reduce the risk of acquisition of HIV by fixed multiplicative factors per sex act, representing their biological efficacy on transmission from infected to uninfected individuals. The efficacy values are detailed in Tables S2-5. Individuals infected whilst using different products are assumed to have the same infectiousness as others. Effective coverage (representing both usage and adherence) of each intervention can be targeted by age or risk group. Intervention scale-up is linear over a fixed period of time, and the target coverage is maintained thereafter.

Table S2. Intervention assumptions for male condoms.

Partnership types are described by both participants; in case of conflict, we use the higher value for condom use from the two. Where a range of values are given, coverage increases from the lower to the higher value in the period 1995-2008 due to behaviour change.

| Partnership type | Efficacy | Current coverage | Effective coverage  (number of sex acts with condom / total sex acts) | | Coverage increase starts | Duration for coverage change |
| --- | --- | --- | --- | --- | --- | --- |
|  |  |  | Constant Coverage baseline | Projected Scale-up baseline |  |  |
| Female sex worker <> Client | 90% [18] | Informed by data and in model calibration | 0.8 - 29% | 60% | 2019 | 5 |
| High-risk women, 15-30 years <> Anyone |  |  | 0.4 - 28% | 0.4 - 28% |  |  |
| Low-risk women, 15-30 years <> Anyone |  |  | 0.4 - 28% | 0.4 - 28% |  |  |
| Women, 30+ years <> Anyone |  |  | 0.4 - 28% | 0.4 - 28% |  |  |
| High-risk men <> Low-risk women |  |  | 0.4 - 28% | 0.4 - 28% |  |  |
| High-risk men <> High-risk women |  |  | 0.4 - 28% | 0.4 - 28% |  |  |
| Low-risk men <> Anyone |  |  | 0.2 - 14% | 0.2 - 14% |  |  |
| Low-risk men <> Low-risk women (serodiscordant couples) |  |  | 0.2 - 14% | 0.2 - 14% |  |  |

The condom efficacy estimate represents consistent users.[18] This is higher than the most recent Cochrane review (~80% effectiveness) but that estimate is covers all condom users rather than consistent users only.[19]

Table S3. Intervention assumptions for voluntary medical male circumcision (VMMC).
The model simulates the minimum sufficient circumcision operations required in order to induce at least these levels of coverage.

| Population group | Efficacy | Current coverage | Effective coverage  (number of circumcised men / number of men) | | Coverage increase starts | Duration for coverage change |
| --- | --- | --- | --- | --- | --- | --- |
|  |  |  | Constant Coverage baseline | Projected Scale-up baseline |  |  |
| High-risk men, 15-30 years | 60% [20-22] | Informed by data and in model calibration | 43% | 60% | 2019 | 5 |
| Low-risk men, 15-30 years |  |  |  | 60% |  |  |
| High-risk men, 30-49 years |  |  | 10% | 15% |  |  |
| Low-risk men, 30-49 years |  |  |  | 15% |  |  |

Table S4 Intervention assumptions for early ART
Early ART is defined as >350 CD4 cells per ml^3^.

| Population group | Efficacy | Current coverage | Effective coverage  (number of people that can receive early ART early / total number of HIV-positive people) | | Coverage increase starts | Duration for coverage change |
| --- | --- | --- | --- | --- | --- | --- |
|  |  |  | Constant Coverage baseline | Projected Scale-up baseline |  |  |
| Female sex workers | 85% [9] | 40% | 40% | 60% | 2019 | 2 |
| High-risk women, 15-30 years |  |  | 40% | 60% |  |  |
| Low-risk women, 15-30 years |  |  | 40% | 60% |  |  |
| High-risk women, 30-49 years |  |  | 40% | 60% |  |  |
| Low-risk women, 30-49 years |  |  | 40% | 60% |  |  |
| High-risk men, 15-49 years |  |  | 40% | 60% |  |  |
| Low-risk men, 15-49 years |  |  | 40% | 60% |  |  |

The efficacy estimate includes the assumption that up to 90% of users will be virally suppressed.

Table S5. Intervention assumptions for oral PrEP
Coverage is defined as the coverage of “good users” benefiting from the efficacy values, which takes into account levels of adherence. Wastage of PrEP through temporary provision to bad users is assumed to be incorporated into the average unit costs of PrEP.

| Population group | Efficacy | Current coverage | Effective coverage  (number of adherent users / total number of people) | | Available from | Duration for coverage change |
| --- | --- | --- | --- | --- | --- | --- |
|  |  |  | Constant Coverage baseline | Projected Scale-up baseline |  |  |
| Female sex workers | 90% [23] | 0% | 0% | 10% | 2019 | 4 |
| High-risk women, 15-30 years |  |  |  | 5% |  |  |
| Low-risk women, 15-30 years |  |  |  | 0% |  |  |
| High-risk women, 30-49 years |  |  |  | 0% |  |  |
| Low-risk women, 30-49 years |  |  |  | 0% |  |  |
| High-risk men, 15-49 years |  |  |  | 0% |  |  |
| Low-risk men, 15-49 years |  |  |  | 0% |  |  |

Oral PrEP efficacy is the per-sex act value for good adherers, derived from analysis of individuals in the Partners PrEP study with tenofovir concentrations >40ng/mL.[23] The proportion of each sub-group of the population assumed to be “good adherers” is incorporated into the coverage assumption.

**Supplementary results**

Figure S9. Projected HIV incidence among adults aged 15-49 years from 2020-2050 at different coverage levels.
A. Constant Coverage baseline, 1% CAB-LA coverage among each population group.
B. Projected Scale-up baseline, 1% CAB-LA coverage among each population group.
C. Constant Coverage baseline, 5% CAB-LA coverage among each population group.
D. Projected Scale-up baseline, 5% CAB-LA coverage among each population group.
E. Constant Coverage baseline, 10% CAB-LA coverage among each population group.
F. Projected Scale-up baseline, 10% CAB-LA coverage among each population group.
G. Constant Coverage baseline, 20% CAB-LA coverage among each population group.
H. Projected Scale-up baseline, 20% CAB-LA coverage among each population group.
All figures assume 90% efficacy for CAB-LA.

**
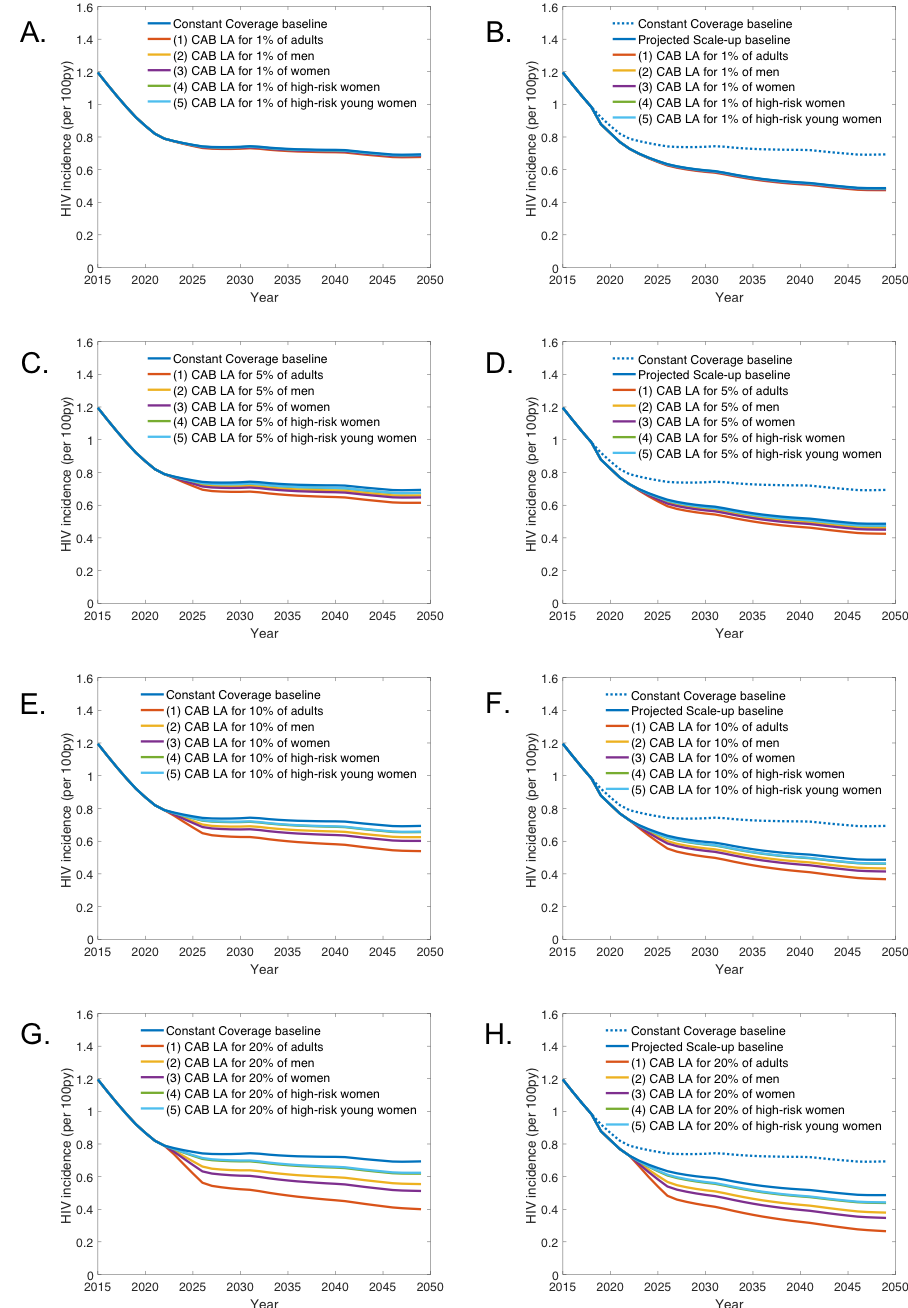
**

Figure S10. HIV infections averted (%) among adults aged 15-49 years from 2023-2050 under different efficacy assumptions and mean duration of use for CAB LA.
A. 1% CAB-LA coverage.
B. 5% CAB-LA coverage.
C. 10% CAB-LA coverage.
D. 20% CAB-LA coverage.
All figures assume that CAB-LA is introduced to the Projected Scale-up baseline.

**
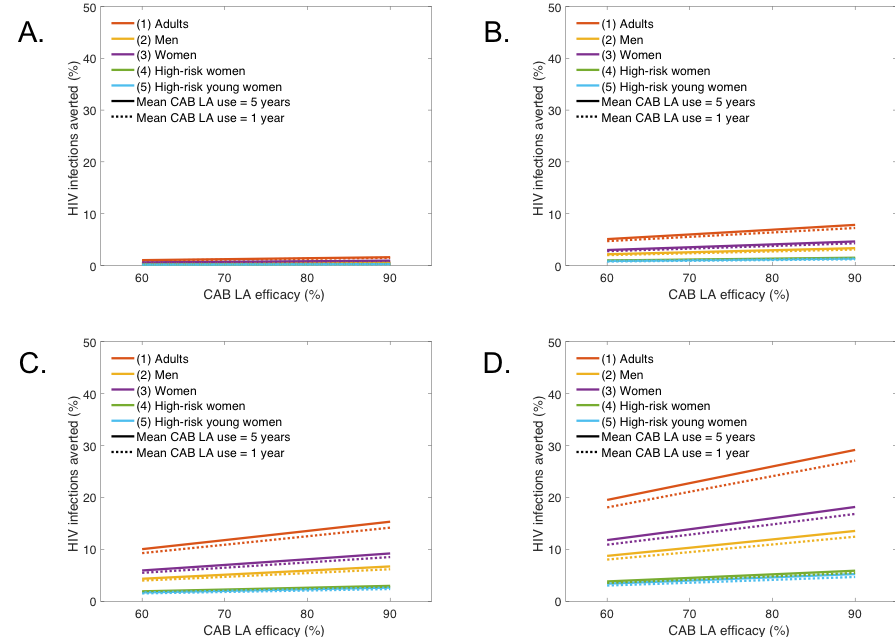
**

Figure S11. The number of person-years of long-acting cabotegravir required to avert one HIV infection.
Calculation performed varying the effective coverage in each population group.
A. 1% CAB-LA coverage, Constant Coverage baseline.
B. 1% CAB-LA coverage, Projected Scale-up baseline.
C. 20% CAB-LA coverage, Constant Coverage baseline.
D. 20% CAB-LA coverage, Projected Scale-up baseline.
All figures assume that CAB-LA is 90% efficacious.

**
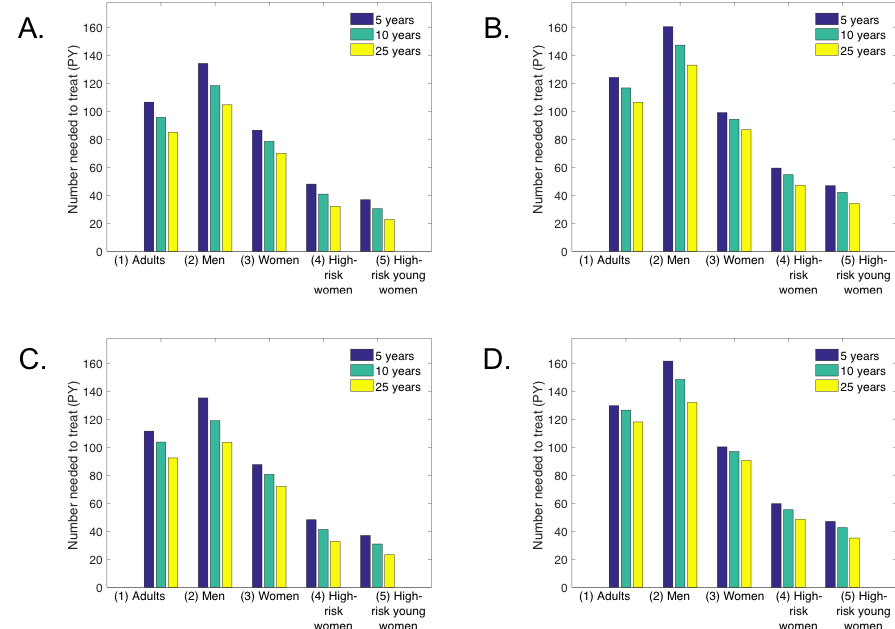
**

**References**

1. Anderson R, May R. Infectious diseases of humans: dynamics and control. Oxford, **1991**.

2. Garnett G, Anderson R. Factors controlling the spread of HIV in heterosexual communities in developing countries: patterns of mixing between different age and sexual activity classes. Philosophical Transactions of the Royal Society B: Biological Sciences **1993**; 342:137-59.

3. Garnett G, Anderson R. Sexually transmitted diseases and sexual behaviour: insights from mathematical models. Journal of Infectious Diseases **1996**; 198:687-93.

4. Hollingsworth T, Anderson R, Fraser C. HIV-1 transmission by stage of infection. Journal of Infectious Diseases **2008**; 198:687-93.

5. Human Sciences Research Council (HSRC). The Fifth South African National HIV Prevalence, Incidence, Behaviour and Communication Survey, 2017: HIV Impact Assessment Summary Report. Cape Town, **2018**.

6. Boily M-C, Baggaley R, Wang L, et al. Heterosexual risk of HIV-1 infection per sexual act: systematic review and meta-analysis of observational studies. The Lancet Infectious Diseases **2009**; 9:118-29.

7. Donnell D, Baeten J, Kiarie J, et al. Heterosexual HIV-1 transmission after initiation of antiretroviral therapy: a prospective cohort analysis. The Lancet **2010**; 375:2092-8.

8. UNAIDS. AIDSInfo. Accessed 13th January 2020.

9. Cohen MS, Chen YQ, McCauley M, et al. Prevention of HIV-1 Infection with Early Antiretroviral Therapy. New England Journal of Medicine **2011**; 365:493-505.

10. Shisana O, Rehle T, Simbayi LC, et al. South African National HIV Prevalence, Incidence and Behaviour Survey, 2012. In: Press H, ed. Cape Town, **2014**.

11. Department of Health, Medical Research Council, OrcMacro. South Africa Demographic and Health Survey 2003. Pretoria: Department of Health, **2007**.

12. Actuarial Society of South Africa. ASSA2008 Model, **2011**.

13. THE NATIONAL HIV AND SYPHILIS PREVALENCE SURVEY SOUTH AFRICA 2007. South Africa: National Department of Health, **2008**.

14. Granich RM, Gilks CF, Dye C, De Cock KM, Williams BG. Universal voluntary HIV testing with immediate antiretroviral therapy as a strategy for elimination of HIV transmission: a mathematical model. The Lancet **2009**; 373:48-57.

15. Mossong J, Grapsa E, Tanser F, Bärnighausen T, Newell M-L. Modelling HIV incidence and survival from age-specific seroprevalence after antiretroviral treatment scale-up in rural South Africa. AIDS (London, England) **2013**; 27:2471-9.

16. Shisana O, Rehle T, Simbayi L, et al. South African National HIV Prevalence, Incidence, Behaviour and Communication Survey, 2008: A turning tide among teenagers? Cape Town, **2009**.

17. National Department of Health (NDoH), Statistics South Africa (Stats SA), South African Medical Research Council (SAMRC), ICF. South Africa Demographic and Health Survey 2016: Key Indicators. Pretoria, South Africa and Rockville, Maryland, USA: NDoH, Stats SA, SAMRC, and ICF, **2017**.

18. Pinkerton SD, Abramson PR. Effectiveness of condoms in preventing HIV transmission. Social Science & Medicine **1997**; 44:1303-12.

19. Weller SC, Davis-Beaty K. Condom effectiveness in reducing heterosexual HIV transmission. Cochrane Database of Systematic Reviews **2002**; 2002.

20. Auvert B, Taljaard D, Lagarde E, Sobngwi-Tambekou J, Sitta R, Puren A. Randomized, Controlled Intervention Trial of Male Circumcision for Reduction of HIV Infection Risk: The ANRS 1265 Trial. PLoS Med **2005**; 2:e298.

21. Bailey RC, Moses S, Parker CB, et al. Male circumcision for HIV prevention in young men in Kisumu, Kenya: a randomised controlled trial. The Lancet **2007**; 369:643-56.

22. Gray RH, Kigozi G, Serwadda D, et al. Male circumcision for HIV prevention in men in Rakai, Uganda: a randomised trial. The Lancet **2007**; 369:657-66.

23. Donnell D, Baeten JM, Bumpus NN, et al. HIV Protective Efficacy and Correlates of Tenofovir Blood Concentrations in a Clinical Trial of PrEP for HIV Prevention. JAIDS Journal of Acquired Immune Deficiency Syndromes **2014**; 66:340-8.
